# Effectiveness evaluation of an integrated automatic termomechanic massage system (SMATH^® ^system) in non-specific sub-acute and chronic low back pain - a randomized double-blinded controlled trial, comparing SMATH therapy versus sham therapy: study protocol for a randomized controlled trial

**DOI:** 10.1186/1745-6215-12-216

**Published:** 2011-10-04

**Authors:** Paolo Buselli, Roberto Bosoni, Gabriella Busè, Paola Fasoli, Elide La Scala, Rita Mazzolari, Federica Zanetti, Sara Messina

**Affiliations:** 1 Hospital "Istituti Ospitalieri di Cremona" Physical and Rehabilitation Medicine Department, Cremona, Italy and Physiotherapy Faculty, University of Brescia, Italy; 2 Hospital "Istituti Ospitalieri di Cremona" Physical and Rehabilitation Department, Cremona, Italy; 3 School of Specialities in Physical Medicine and Rehabilitation, University of Pavia, Pavia, Italy

## Abstract

**Background:**

Low back pain (LBP) is a major health problem in modern society, with 70-85% of the population experiencing LBP at some time in their lives. Each year, 5-10% of the workforce misses work due to LBP, most for less than 7 days. Almost 10% of all patients are at risk of developing chronic pain and disability. Little clinical evidence is available for the majority of treatments used in LBP therapy. However, moderate evidence exists for interdisciplinary rehabilitation, exercise, acupuncture, spinal manipulation, and cognitive behavioral therapy for subacute and chronic LBP. The SMATH^® ^system (system for automatic thermomechanic massage in health) is a new medical device (MD) that combines basic principles of mechanical massage, thermotherapy, acupressure, infrared therapy, and moxibustion. SMATH^® ^is suitable for automatic multidisciplinary treatment on patients with non-specific sub-acute and chronic LBP.

**Methods/design:**

This paper describes the protocol for a double-blinded, sham-controlled, randomized, single-center short term clinical trial in patients with non-specific sub-acute and chronic LBP aged 18 to 70 years. The primary outcome will be the effectiveness of SMATH^® ^versus sham therapy (medical device without active principles) determined by evaluating self perceived physical function with Roland Morris Disability Questionnaire (RMDQ) scores after 4 weeks of treatment (end of treatment). Major secondary outcome will be effectiveness of SMATH^® ^determined by evaluating self perceived physical function comparing RMDQ scores between end of treatment and baseline. The trial part of the study will take 7 months while observational follow-up will take 11 months. The sample size will be 72 participants (36 for each arm). The project has been approved by the Ethical Committee of Cremona Hospital, Italy on 29 November 2010.

**Discussion:**

Compared to other medical specialties, physical and rehabilitation medicine (PRM) has not yet received the deserved recognition from clinicians and researchers in the scientific community, especially for medical devices. The best way to change this disadvantage is through well-conducted clinical research in sham-controlled randomized trials. Sham treatment groups are essential for improving the level of evidence-based practice in PRM. The present trial will counter the general lack of evidence concerning medical devices used in LBP therapy.

**Trial Registration:**

ISRCTN: ISRCTN08714168

## Background

Low back pain (LBP) is a major health problem in modern society; 70-85% of the population will experience LBP at some time in their lives [1]. Each year, 5-10% of the workforce misses work due to LBP, the majority for less than 7 days [2]. Almost 10% of all patients are at risk of developing chronic pain and disability, accounting for more than 90% of the costs associated with back-related disability [3]. LBP incurs substantial treatment and productivity costs worldwide [4]. Healthcare and social costs for LBP are increasing rapidly. In the United States, healthcare costs among people with LBP increased 65% between 1997 and 2005, more rapidly than overall healthcare costs [5]. In the United States, chronic LBP is estimated to cost Americans over 70 billion dollars per year in health care expenditures [6]. The total cost of treatment, lost work days, and disability due to chronic pain in 1995 and 1996 was estimated to be between 150 and 215 billion dollars [7]. In the UK, 1.3 million people receive physiotherapy for LBP each year, with a global cost of £150 million for the National Health System (NHS) [8]. In Italy, the global economic impact of LBP is approximately €24 billion per year, and patients pay almost all fees.

LBP has been difficult to treat solely using medical interventions due to the complex interplay of biological, psychological, and social factors in its onset and persistence. LBP is usually a benign and self-limiting condition, but many patients look for some type of therapy to relieve their symptoms and provide hope for a cure. For this reason, more than 50 potential therapies promise to relieve the pain [2,9], lessen the suffering, and offer a cure for this problem. However, sound evidence exists for a small number of these therapies [9]. For the interventions with multiple reviews, conflicting conclusions have been reached about effectiveness due to the heterogeneity and poor quality of the trials, but there are more qualitative systematic reviews than meta-analyses, and two-thirds of systematic review authors emphasize that more high quality trials are needed [10]. Despite advances in knowledge, technology, and procedures, no medical treatment has been demonstrated to consistently and completely alleviate LBP. Clinical evidence for the majority of treatments used in LBP therapy is also inconsistent. However, moderate evidence exists for interdisciplinary rehabilitation, exercise, acupuncture, spinal manipulation, and cognitive behavioral therapy for sub-acute and chronic LBP [5,11]. In particular, multidisciplinary interventions seem to be the most promising approach for patients with sub-acute and chronic LBP [12]. Intensive multidisciplinary biopsychosocial rehabilitation with a functional restoration approach has been shown to reduce pain and improve function in patients with chronic LBP [13]. In addition, empirical evidence has shown the sort of information and advice that should be given to patients with back pain, confirming that such information and advice may be a potent element of the health care intervention [14]. Soft-tissue massage is thought to improve physiological and clinical outcomes by offering symptomatic pain relief through physical and mental relaxation and increasing the pain threshold via the release of endorphins [2,15]. The gate-control theory predicts that massaging a particular area stimulates large diameter nerve fibers. These fibers have an inhibitory electrical input to T cells, which are the first cells to project into the central nervous system within the spinal cord [2]. T-cell activity is likely depressed, whereas small diameter nerve fibers (nociceptive fibers) have an excitatory input, and pain relief follows [16]. Massage therapy may provide benefits by shifting the autonomic nervous system from a state of sympathetic response to a state of parasympathetic response. Massage is similar or superior to other conservative therapies, such as exercises, mobilization, relaxation, physical therapy, acupuncture, and self-care education [2]. Massage is beneficial for patients with sub-acute and chronic nonspecific LBP in terms of improving symptoms and function, but the benefits of massage increase when combined with exercises and education [2]. Massage has powerful analgesic effects if applied to acupuncture points, a technique known as "acupressure" [2]. Two studies that compared massage to inert treatment (sham therapies) showed that massage is superior for pain and function in both short and long-term follow-up [17,18]. No serious adverse events were reported by any patient in the studies reviewed [2]. Massage alone is unlikely to be cost-effective [5]. Focusing on high-quality randomized controlled trials (RCTs) with a sufficient sample size to draw firm conclusions is particularly recommended for future research involving LBP therapies [12].

## Trial Rationale

SMATH^® ^is a new medical device that combines basic principles of mechanical massage, thermotherapy, acupressure, infrared therapy, and moxibustion. SMATH^® ^is included in the "conservative treatments" category [13]. This device is capable of releasing modulated and controlled thermomechanic energy on the patient following a programmable and fully reproducible automatic treatment program selected by the operator. Energy (thermomechanic and infrared) is released at the same time under the spine and legs of the patient. We think this system provides a true automatic multidisciplinary treatment for patients with sub-acute and chronic LBP. The SMATH^® ^system should theoretically be capable of combining the single clinical benefits of massage, thermotherapy, infrared, and acupressure, removing the dependency on operator capability, though obviously missing some elements of human touch, and providing maximum automaticity and reproducibility and producing a real clinical added value for patients. The literature involving different reviews show that little clinical evidence exists on the use of these endorsed therapies on their own. In particular, there is little evidence for massage performed by hands or a mechanical device, or and for superficial heat release [12]. No evidence is available for thermotherapy [13], or for moxibustion, infrared therapy, and acupressure. On the other hand, moderate evidence indicates the effectiveness of multidisciplinary treatment compared to other kinds of active treatment in regards to pain intensity at short-term follow-up [12]. The SMATH^® ^system was developed in an attempt to create an effectiveness advantage by combining different basic theoretical principles. The first clinical results, from the safety clinical study involving 46 patients inside the process to obtain the European Conformity (CE) mark for SMATH^®^, showed the following: RMDQ average score of 10.96 (sd = 3.04; p < 0.05) at baseline and 3.21 (sd = 2.99; p < 0.05) after 3 months, and the average quality adjusted life year (QALY) was 0.46 (sd = 0.13; p < 0.05) at baseline and 0.81 (sd = 0.12; p < 0.05) after 3 months. These data have not been published because they were not suitable for publication and represented the results obtained by one clinical study in which the primary outcome was the demonstration of clinical safety. The internal clinical report concerning this study has been delivered to the notified body in charge of the CE mark process. The SMATH^® ^system obtained the CE mark on 20 December 2010. The current RCT was designed to investigate and test the SMATH^® ^system for use in LBP therapy.

The cost/effectiveness ratio for the SMATH^® ^system may also be interesting and could play an important role in the Italian NHS strategy for LBP interventions. An HTA procedure has already been launched in Italy for this new therapy. Like all new technologies and interventions, the clinical effectiveness of the SMATH^® ^system has to be proven by clinical studies in a PRM context. Compared to other medical specialties, PRM has not yet received the recognition deserved from clinicians and researchers in the scientific community, especially for medical devices [19]. We think this unfavorable scenario is due to several reasons, one of which is the lack of high-quality clinical trials in the PRM field. Conducting double-blind placebo-controlled trials in the specialty is difficult; the majority of studies in PRM are practically based on clinical observation, uncontrolled observational studies, or clinical trials comparing two different active therapies. Consequently, these studies are not suitable for providing sufficient proof of efficacy for these therapeutic modalities. The only scientifically recognized way to achieve this goal is to design and manage high-quality double-blind placebo-controlled trials. Sham treatment groups in clinical trials are essential for improving the level of evidence-based practice in PRM. This trial was conceived to reduce the lack of evidence and quality for LBP therapy. No major and minor adverse events or adverse effects have been observed during the use SMATH^® ^on patients with LBP. The SMATH^® ^system has the CE mark as it has passed all tests concerning safety for the patient, operators, and environment. For this reason, no adverse events are expected during this clinical study. However, if adverse events occur, they will be reported to the regulatory authority and ethics committee in accordance with applicable regulations. Trial results will be used to inform HTA procedure already involving SMATH^® ^therapy for health care policies regarding the treatment of LBP in Italy.

## Methods

### Design

**Figure 1:**
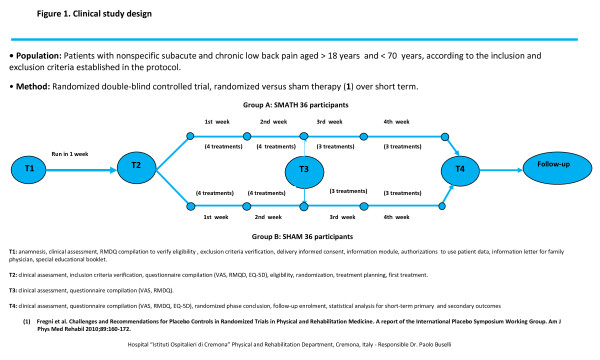
**Clinical Study Design**.

**Figure 2:**
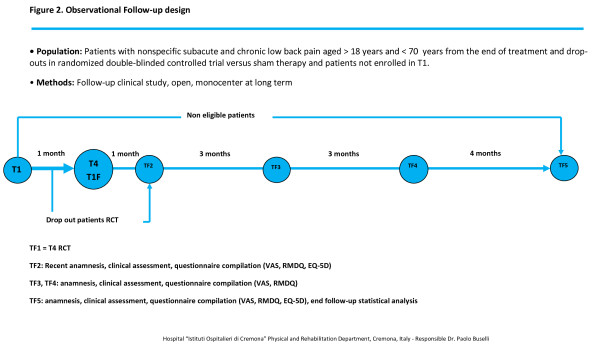
**Follow-up Design**.

This is a double-blinded, sham-controlled, randomized, single-center short term clinical trial involving 72 participants. Thirty-six participants will be treated with SMATH^® ^therapy and compared to 36 participants treated with sham therapy (same medical device without active principles). All participants are patients with non-specific sub-acute and chronic LBP, aged 18 to 70 years. Exclusion criteria verification will be done at baseline (T1) with the identification of eligible participants. One week after T1 (T2), eligible participants who have signed informed consent and satisfy the inclusion criteria will be randomized into either the SMATH^® ^or sham groups. Participants who are not randomized will be submitted to the most suitable therapy by the investigators and sent to TF1 to enter in the observational follow-up. Randomized participants will attend clinic visits at T2 (one week after T1), T3 (two weeks after T2) and T4 (two weeks after T3). The design of the clinical trial is depicted in Figure 1. At T4, treatments will be complete and all participants can continue the most suitable therapy, as indicated by the investigators, and enter the long-term observational follow-up phase, which will last 11 months. All participants will be followed starting at TF1 (same time as T4,) for 11 months and will attend clinic visits at 1 month (TF2), 4 months (TF3), 7 months (TF4), and 11 months (TF5) after TF1. The design of the observational follow-up period is depicted in Figure 2.

### Eligibility

Subjects with sub-acute and chronic non-specific LBP diagnosed according to generally accepted scientific criteria [13] were enrolled. Inclusion criteria were as follows: aged between 18 and 70 years, ability to sign informed consent, ability to complete the study, ability to complete the questionnaires, RMDQ score ≥ 4 at T2 [20], and negative answers on all exclusion questions at T1. The rationale for the inclusion criteria is provided in Table 1.

**Table 1:** Rationale for Inclusion Criteria

Inclusion criteria	Rationale
Age between 18 and 70 years	This age interval was chosen in accordance with actual main reviews in PRM [2,10,12]

Ability to sign informed consent	Mandatory for participant inclusion in clinical studies (Helsinki Declaration article 22)

Ability to complete the study	To prevent drop-outs

Ability to complete the questionnaire	Mandatory for accurate data collection

RMDQ score ≥ 4 at T2	From the literature [20]

Negative answers for all exclusion criteria	This check performed at T2 confirms that the exclusion evaluation was performed at T1.

Exclusion criteria were as follows: pregnancy (real or suspected) or breast-feeding; receiving physical therapy treatments in the 15 days preceding T1; treatment with cortisone in the month preceding T1, implanted with pacemaker or implantable cardioverter defibrillators (ICD) or, more generally, a user of active implantable devices; use of medullar stimulators and infusion pumps; recent or ongoing deep venous thrombosis (DVT); use of spine stabilization devices; serious osteoporosis associated with bone fracture risk, including soft or acutely infected bone tissues; acute cardiovascular disease; neoplastic disease; systemic rheumatic disease; or traumatic spinal episodes in the 3 months preceding T1. The rationale for the exclusion criteria is provided in Table 2.

**Table 2:** Rationale for exclusion criteria

Exclusion criteria	Rationale
Pregnancy (real or suspected) and breast-feeding	These patients have a risk of some alteration due to the mechanical and thermal energy release

Subjects who received physical therapy treatments in the 15 days preceding T1	These treatments could influence the results of the study

Subjects treated with cortisone in the month preceding T1	This treatment could influence the results of the study

Subjects implanted with pacemakers and ICDs (implantable cardioverter defibrillators); more generally, users of active implantable devices	In order to prevent possible electromagnetic interference between SMATH^® ^and the implantable device, which is potentially dangerous for the patient (cautelative criteria)

Subjects with medullar stimulators and infusion pumps with recent or ongoing DVT (deep venous thrombosis)	To prevent possible electromagnetic and mechanical interference between SMATH^® ^and the implantable device, which is potentially dangerous for the patient (cautelative criteria)

Subjects with spine stabilization devices	These treatments could influence the results of the study

Subjects with serious osteoporosis associated with bone fracture risk	Mechanical energy release on these patients could increase bone fracture risk

Subjects with logically soft or acute infection of bone tissues	Thermal and mechanical energy release could increase infection diffusion

Subjects with acute cardiovascular disease	These patients need to be submitted to specific clinical evaluation before being treated for LBP

Subjects with neoplastic disease	These patients need to be submitted to specific clinical evaluation before being treated for LBP

Subjects with systemic rheumatic disease	These patients need to be submitted to specific clinical evaluation before being treated for LBP.

Subjects with traumatic spinal episodes the 3 months preceding T1	Mechanical energy release on these patients could be dangerous.

### Setting and locations where the data will be collected

A special area inside the Head Investigator's office in the Hospital will be dedicated to data collection and storage. Case report forms (CRFs) for the enrolled subjects will be kept strictly inside this area. Treatment plan sheets on which the treatment plan for each randomized participant will be recorded will be delivered externally by the technician in charge of executing the treatment. Before randomization, randomization envelopes will be held in this area by the Head Investigator and remain sealed, even after delivery, until used by the technician in charge of administering the treatment. Access to the CRFs by the investigators and monitor will be authorized by the Head Investigator. The technicians in charge of administering treatment will be responsible for storing the envelopes and treatment plan after randomization and treatment plan; they will have to practice maximum confidentiality about their contents as investigators will not have access to this information.

### Randomization

A simple randomization process has been conceived through three different steps according to the Consolidated Standards of Reporting Trials (CONSORT) statement: sequence generation, allocation concealment, and implementation.

**Sequence generation **has been achieved through a "congruential multiplier" statistical algorithm application, using IBM Statistical Package for Social Sciences (SPSS) Version 19 [21,22] and was stratified with 1:1 allocation random block size of 36. The randomization list reports a progressive randomization number for randomized participants (from 1 to 72), and the treatment (A or B) will be assigned for each of them.

For **allocation concealment**, the randomization list was produced in three numbered copies. The first copy was delivered to the Ethics Committee inside an envelope sealed with warm sealing wax. A second copy was delivered to the Head Investigator in a similar sealed envelope. The Head Investigator will save his copy in a locked box in his office. He can open the envelope in the case an emergency arises. A third copy has been kept by the monitor of the study who managed the sequence generation. The monitor also prepared 72 opaque red envelopes containing randomization sheets. On each envelope is a number (from 1 to 72) and space for the patient's name and date of birth. The randomization sheet (opaque red) reports the progressive randomization number and assigned treatment (A or B) based on the randomization list. All of the envelopes have been closed and sealed with warm sealing wax. The 72 randomization envelopes were delivered to the Head Investigator, who will keep them in a locked box in his office. At the time of randomization, the investigator who decides to randomize the participant will take the first of the envelopes from the box in order of sequence and write the participant's name and date of birth on the envelope (to prevent subversion of the allocation sequence as suggested by Consort 2010, Item 9). Immediately after, the investigator will deliver the closed envelope and intervention plan to the technician in charge of administering the treatment. The technician will open the envelope to start the intervention, saving the envelope and randomization sheet in a locked box. The technicians involved in this phase of the study have been trained and educated to maintain maximum confidentiality concerning allocation concealment. This method will provide double-blinded conditions. Clinical investigators who enroll and evaluate participants will not know which treatment has been assigned to them.

**Figure 3:**
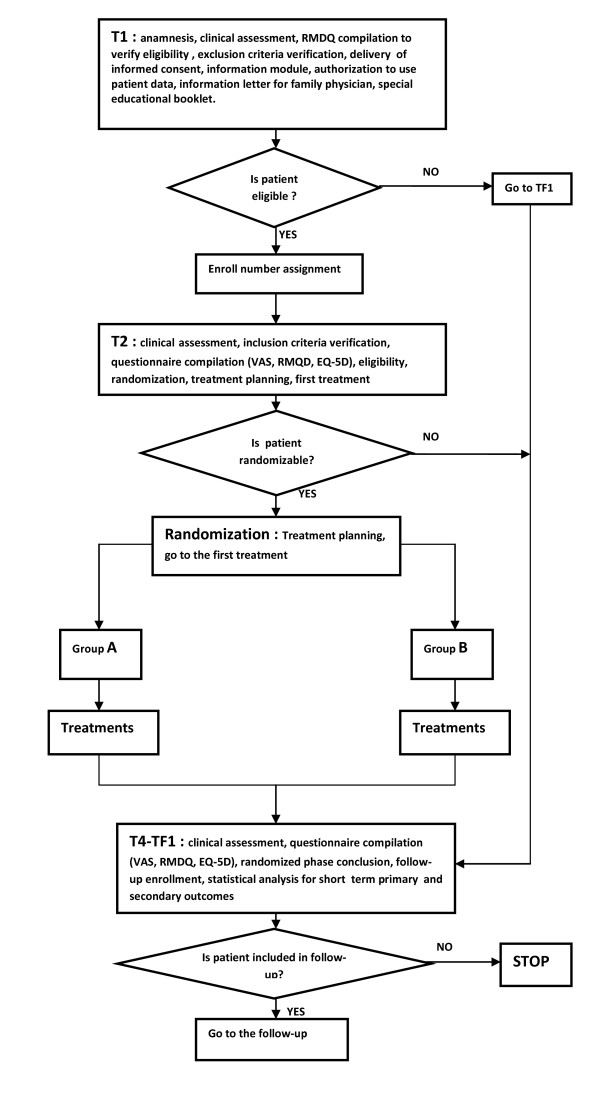
**Randomization Implementation Process**.

**Implementation **of the randomization process will conform with the study requirements and steps described above. The process involves participant eligibility analysis, obtaining patient information, enrollment, treatment assignment, and data analysis to avoid any kind of bias. The implementation process is depicted in Figure 3.

### Interventions for each group

Participants assigned to the SMATH^® ^therapy group (A arm) will have 14 treatment sessions of 45 minutes over 4 weeks starting at T2. The treatment module is associated with a dedicated educational booklet for each patient that provides general and behavioral indications for preventing LBP. In this clinical study, the educational booklet will be delivered to all enrolled participants to help them correct their lifestyle during and after the intervention period. In this dedicated booklet, different sections explain to the participants spine anatomy, LBP, LBP prevention, advantages of physical activity, suggestions for positions for walking, sleeping, and to keep weight under control, and exercises to prevent LBP.

During the first and second week (from T2 to T3), 4 sessions will be done per week, whereas 3 sessions per week will be planned for the third and fourth week (from T3 to T4). The SMATH^® ^system will be set-up with treatment variables (warm-up time, automatic treatment program, active element temperature, infrared insertion) conforming to the scheduled treatment data sheet delivered to the technician after randomization. Sham therapy will be implemented using a dedicated SMATH^® ^device. This medical device, which was conceived and manufactured exclusively for this clinical study, has the same external look and design of the SMATH^® ^device. The active principles (thermomechanic and infrared energy release) have been fully blocked in the sham device, but the participants' perceptions will be more or less the same (warm, vibration, noise). The engineering department has made important efforts to create a device with the appearance of the active machine, inducing feelings similar to those of the real device [19]. This effect has been obtained by applying some technical solutions without releasing any active energy. Thermal perception is generated using special lamps with different wavelengths than the real SMATH^® ^device with 90% less thermal power (just to induce the feeling of heat). Infrared emission has been removed, as well as the active rollers that release mechanical energy to the back and legs, replaced by an inert, flat wood plate. The sham device will be set-up with the same treatment variables as the SMATH^® ^device. The noise and vibration generated by the sham device will be the same as that of the SMATH^® ^system because the devices have the same mechanical configuration, including the same motor and transmission equipment, operating in the same mode. Despite this similarity, a therapeutic placebo effect could be introduced by the sham device. This risk has been considered and accepted as a natural risk of the RCT with a sham control.

**Figure 4:**
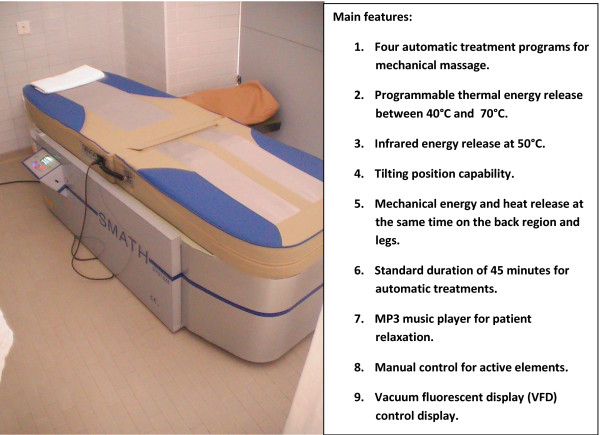
**The SMATH^® ^System**.

**Figure 5:**
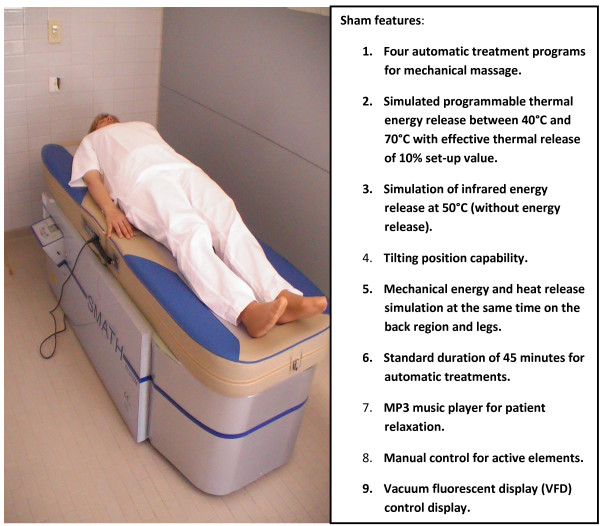
**The SMATH^® ^sham version**.

Participants assigned to the sham therapy arm will receive the same treatment module scheduled for the active SMATH^® ^arm (14 sessions in four weeks from T2 to T4). The participants in the sham arm will also receive the educational booklet. The SMATH^® ^and sham devices are located inside two different therapy boxes and treatment will be given at separate times to prevent contact between participants in the different treatment arms. The operators managing treatments will be the same for the SMATH^® ^and sham devices, and they have been trained to answer the participants' questions about the procedures. The same operators will also be trained and educated in keeping participants in the study, avoiding an increased drop-out rate. The main features of the SMATH^® ^system and sham version are presented in Figures 4 and 5, respectively.

## Discussion

### Outcomes

This clinical study was designed to include the following general outcome measures: pain, overall improvement, back-specific functional status, well being (quality of life), and disability. The questionnaires that will be used to collect data from the participants are: visual analog scale (VAS) for pain [12], Roland Morris Disability Questionnaire (RMDQ) for effectiveness, specifically back-specific disability [12], and Euro Qol-5D questionnaire (EQ-5D) for quality of life, which is one of the most used and suitable questionnaires for QALY calculation [5]. These questionnaires were chosen by considering the major systematic reviews regarding physical and rehabilitory interventions for sub-acute and chronic non-specific LBP [2,4,9,10,12,15]. Participants will complete the VAS at T2, T3, T4, TF1, TF2, TF3, TF4, and TF5; RMDQ at T1, T2, T3, T4, TF1, TF2, TF3, TF4, and TF5; and EQ-5D at T2, T4, TF1, TF2, and TF5.

### Primary outcome

The primary outcome of the trial is represented by the clinical effectiveness of the SMATH^® ^system versus sham therapy after 4 weeks of treatment in patients with sub-acute and chronic non-specific LBP, determined by evaluating self perceived physical function with RMDQ scores. The status of physical function in participants treated with SMATH^® ^interventions will be compared with the status achieved in participants in the sham control arm at the end of treatment (T4) [12].

### Secondary outcomes

The secondary outcomes of the trial are: 1) Effectiveness of SMATH^® ^determined by evaluating self perceived physical function comparing RMDQ scores between end of treatment and baseline. 2) Pain perception change from baseline (T2) to end-treatment (T4), evaluated by VAS scores. 3) Quality of life at different steps of the study (T2 and T4), evaluated by EQ-5D scores (QALY). 4) Verified general feasibility of the medical device compared to sham therapy in terms of methodology, sample size, and drop-out rate, among other measures, to constitute a real pilot study and act as a possible reference for future generations of RCTs involving the medical device, conforming to the evidence-based medicine (EBM) criteria for LBP therapy [13] according to the CONSORT statement based on new methodological evidence and to the International Placebo Symposium Working Group recommendations [19].

### Observational follow-up and duration of the study

Observational follow-up will start at TF1 (T4) and cover 11 months. During this period, all of the participants will come to the treatment centre to attend clinic visits one month after TF1 (TF2), 3 months after TF2 (TF3), 3 months after TF3 (TF4), and 4 months after TF4 (TF5). Figure 2 shows the design of the observational follow-up period. The estimated study duration for enrollment, execution and observational follow-up will be 18 months.

### Assessment of outcomes

Four steps of participant examination are planned in this RCT (T1, T2, T3, and T4); during which, general clinical data will be collected about the persistence of LBP symptoms, previous treatment, morphological data, patient activity, blood chemistry tests, anamnesis, and eventual clinically important imaging investigations that were recently done. The effectiveness of SMATH^® ^versus sham will be determined by evaluating self-perceived physical function with RMDQ scores at T4. The secondary outcome of SMATH^® ^therapy effectiveness will be determined by evaluating improvements in patients' motion functionality between T2 and T4 using RMDQ scores. The evolution of pain perception during the study will be measured by VAS scores submitted by the participants at T2, T3, and T4. Quality of life for participants will be estimated by the comparing the EQ-5D questionnaires submitted at the beginning and end of treatment (T2 and T4). During the observational follow-up period all participants will be examined at TF1, TF2, TF3, TF4, and TF5 for general patient status: anamnesis, blood pressure, heart rate in beats per minute, and morphological variables (height, weight, Body Mass Index [BMI]). The RMDQ and VAS questionnaires will be given on the participants at TF1, TF2, TF3, TF4, and TF5, and EQ-5D will be given at TF1, TF2, and TF5. All of the data collected during the study and follow-up will be reported to the CRF.

### Sample size calculation

Due to the difficulty of finding solid references in the literature regarding the probability of success for different arms of RCTs comparing therapy with medical devices versus sham therapy [19], some basic statistical assumptions have been made in regards to the level of inhibition technically introduced into the SMATH^® ^active principles to obtain a sham device. The therapeutic effect induced by the sham device has been estimated to be 10% of the effect induced by the SMATH^® ^system. Consequently, the probability of SMATH^® ^therapy success has been fixed at 50% (P_1_) [2], whereas a probability of 10% (P_2_) has been fixed for success with sham therapy. Thus, the success difference between the two study arms (P_1 _- P_2_) is 40%. This important delta has been fixed to be statistically significant also in the case of unexpected placebo effect of sham. The false-positive risk factor α (type I error rate) has been fixed at 0.05 (5%). The probability of discovering a difference between the two study arms has been fixed at 90% (study's power). Based on the study's power, (1 - β) = 0.9, the false-negative risk factor rate β is 0.1 (10%). The value of the function *f *(α, β) was calculated by Geigy's tables [23]. Thus, the sample size in each arm of the study (n) should be n = 26. Drop-out has been considered to prevent having an insufficient sample at the end of the study due at the high risk of drop-out of participants in sham arm. A drop-out rate of 15% (0.15) has been estimated based on the literature concerning RCT versus sham in PRM [19]. The sample adjusting factor μ was calculated using the formula μ = 1/(1 - R)^2 ^[24-26], where R represents the drop-out rate. Therefore, μ = 1/(1 - 0.15)^2 ^= 1.3841, and the sample size after correcting for drop-out will be:n# = nμ = 36. The sample of each group will be 36 participants for a total study group of 72 subjects.

### Statistics and quality

The accuracy and completeness of the collected and registered data will be checked daily by clinical investigators to provide a suitable level of quality for the study. The external monitor will be responsible for arranging random periodic tests of quality throughout the study. In particular, controls will involve the quality and completeness of the CRFs, the treatment execution modalities, the drop-out rate, and correct respect of enrollment criteria application. Data analysis will be conducted using IBMSPSS 19 [21,22]. The morphological and clinical status of participants will be analyzed statistically. Motion functionality, level of pain perception, and quality of life will be evaluated through statistical analysis of the questionnaire scores. Short-term results will be evaluated to elaborate VAS and RMDQ scores, and medium and long-term results will be evaluated by EQ-5D scores, which will also be used to calculate the QALY and incremental cost effectiveness ratio (ICER) index [5,22], which is the most used and suitable index for analyzing the cost/effectiveness of PRM therapies [5]. Pain perception measured by VAS in the different phases and its severity analysis will be evaluated by analysis of variance using SPSS 19. Event incidence (SMATH^® ^versus sham) will be estimated using Kaplan-Meier curves, which will be compared using the log-rank test. Multivariable analysis will be performed using the Cox model.

### Patient information material

Correct involvement and informed participant processes are key issues for the success and credibility of the study. Clinical investigators will be responsible for informing participants and involving them in the most appropriate way. This goal will be pursued by discussing the study objectives, risks, benefits, and modalities with participants at T1. Each participant will make a decision as to whether they will participate in the study, which will have to be clearly checked by investigators and documented. In this study, clinical investigators will submit and explain to each participant the following documents: (1) signed informed consent, (2) information module, (3) authorization to use personal sensitive data based on privacy law, (4) information letter that will be delivered to the family physician, (5) the dedicated educational booklet. This booklet must be considered a part of the SMATH^® ^treatment module.

### Adverse events

Adverse events will be managed to conform with good clinical practice and actual normative rules, **listed in **additional file 1. All adverse events will be reported to the regulatory authority and ethics committee in accordance with applicable regulations. The report will specify the types of events, and whether they were device related or non-device related, that shall be reported and the timing for such reporting.

## Protocol changes

Eventual unplanned and important changes to the study protocol involving, for instance, eligibility criteria, interventions, examination data collection, method of analysis, and outcomes, will be submitted to the ethics committee as amendments for approval. In the case of approval and application, they will be reported at the end of the study. All deviations from the protocol during its execution will have to be justified and documented in CRFs for the participants.

## List of abbreviations used

In this manuscript, the following abbreviations have been used:

BMI: Body Mass Index; CRF: Case report form; CE: Conformité Européenne (European Conformity); CONSORT: Consolidated Standards of Reporting Trials; DVT: Deep venous thrombosis; EBM: Evidence-based medicine; EQ-5D: Euro Qol 5D questionnaire; HTA: Health technology assessment; ICD: Implantable cardioverter defibrillator; ICER: Incremental Cost Effectiveness Ratio; LBP: Low back pain; MD: Medical device; NHS: National Health System; PRM: Physical and rehabilitation medicine; QALY: Quality adjusted life year; RCT: Randomized controlled trial; RMDQ: Roland Morris Disability Questionnaire; SMATH^®^: System for automatic thermomechanic massage in health; VAS: Visual analog scale.

## Competing interests

The authors declare that they have no competing interests.

## Authors' contributions

PB acts as guarantor of the data, he has designed the interventions and supervises research staff. PB and SM designed the study, which is funded by internal resources. GB, RB, PF, and ELS will be responsible for data collection and analysis, and they contributed to drafting the paper. PB, with SM, reviewed the manuscript critically. RM and FZ will manage the treatment of randomized participants after randomization. All authors reviewed and edited the draft version of the manuscript and approved the final version. All authors have approved the submission of the present paper to *Trials*.

## Supplementary Material

Additional file 1 **List of the Italian and international laws and norms for clinical studies**. This additional file contains a list of the italian and international laws and norms which have been respected for this randomized double-blinded controlled trial. This clinical study has been designed and will be managed to conform with the following Italian and international laws and norms listed in additional file 1. Click here for file
