# Supplementary material for: Effectiveness evaluation of an integrated automatic termomechanic massage system (SMATH® system) in non-specific sub-acute and chronic low back pain - a randomized double-blinded controlled trial, comparing SMATH therapy versus sham therapy: study protocol for a randomized controlled trial
Source: Trials. 2011 Oct 4;12:216. doi: 10.1186/1745-6215-12-216 (PMC3214170; doi:10.1186/1745-6215-12-216)
Supplement: Additional file 1 — List of the Italian and international laws and norms for clinical studies. This additional file contains a list of the italian and international laws and norms which have been respected for this randomized double-blinded controlled trial. This clinical study has been designed and will be managed to conform with the following Italian and international laws and norms listed in additional file 1. [file 1745-6215-12-216-S1.PDF]

Additional file 1

List of the Italian and International laws and norms for clinical studies

1. UNI EN ISO 9001:2008 Quality Management Systems –Requirements.
2. UNI EN ISO 13485:2004 Medical Devices: Quality Management Systems requirements for regulatory purposes.
3. [Directive 93/42 of CEE Council 14 of June 1993 concerning Medical Devices] (Italian),
4. [D.L. n° 46 24 of February 1997: Fulfillments of Directive 93/42 CEE concerning Medical Devices] (Italian).
5. [D.L. of 20/02/2007. News modalities for fulfillments planned into art. 13 of D.L. 24 of February 1997 n° 46 and following modifications and for active implantable Medical Devices registration as well as for inscription into the Medical Devices Registry] (Italian).
6. [D.L. of 21/12/2009 of Italian Ministry of Health. News modalities for fulfillments planned for active implantable Medical Devices registration as well as for inscription into the Medical Devices Registry] (Italian).
7. [D.L. n° 37 of 25/01/2010 of Italian Ministry of Health. Directive 2007/47/CE fulfillment] (Italian).
8. [MEDDEV 2.12-1 Revision 6 Guidelines concerning Medical Devices technical-vigilance] (Italian).
9. [Italian Ministry Note of 27/07/2004 Vigilance draft] (Italian).
10. UNI CEI ISO 14971:2009 Medical Devices: Application of risk management to medical devices,
11. [D.L. 9 of April 2008, n° 81 “Fulfillment of article 1 of Low 3 of August 2007, n° 123, in terms of health protection and safety protection inside work places”] (Italian).
12. [D.L. 30 giugno 2003, n° 196 “Rules regarding personal data protection”] (Italian).
13. World Medical Association Declaration of Helsinki (1964) 59th WMA General Assembly Seoul October 2008.

14. Convention of Oviedo - European Council 1997.
15. [D.L. 2 of August 2005 of Italian Ministry of Health. Modalities for present documents for Clinical Study with Medical Devices notification] (Italian).
16. [Draft from Italian Ministry of Health 5 of December 2007. Modalities for present documents to inform Ministry of Health about Clinical Investigations with Medical Devices and clarifications concerning Ministry note of 26 of February 2007 regarding post-marketing clinical evaluation with Medical Devices] (Italian).
17. [Note of Italian Ministry of Health DGFDM. VI/6821/P-I.5.i.m.2 of 26 of February 2007. Administrative procedures concerning clinical investigations management involving Medical Devices with CE Mark] (Italian).
18. [D.L. of Italian Ministry of Health 17 of December 2004. General prescriptions and conditions, concerning drugs clinical investigations execution, referring to those oriented to improve clinical practice, as a part of health care] (Italian).
19. [D.L. n° 211 24 of June 2003. "Fulfillment of Directive 2001/20/CE concerning Good Clinical Practice application inside clinical investigations execution for drugs for clinical use"] (Italian).
20. MEDDEV 2.12-2 may 2004. Guidelines on post market clinical follow-up for Medical Devices.
21. MEDDEV 2.7.1 Rev.3. Guidelines on Medical Devices-Clinical evaluation: a Guide for manufactures and Notified Bodies.
22. [CPMP/ICH/135/95 . Guidelines for Good Clinical Practice] (Italian).
23. [D.L. of Italian Ministry of Health 2 of August 2005. Presentation modalities for documents informing about clinical investigations with Medical Devices] (Italian).
24. [D.L. n° 200 6 of November 2007. Fulfillment of Directive 2005/28/CE concerning principles and detailed guidelines for good clinical practice regarding drugs in clinical investigation for human use, as well as requirements for import or manufacturing this kind of drugs] (Italian).

25. UNI EN ISO 14155-1:2009. Clinical investigations, for medical devices for human subjects. Part 1: General requirements.
26. UNI EN ISO 14155-2:2009. Clinical investigations of medical devices for human subjects. Part 2: Clinical investigations plans.
27. SG1/N011:2008. Summary Technical Documentation for demonstrating conformity to the Essential Principles of safety and performance of Medical Devices (STED).
28. SG1-N44:2008. Role of standards in the assessment of Medical Devices.
29. SG1/N029:2005. Information document concerning the definition of the term Medical Device.
30. SG1/N040:2006. Principles of conformity assessment for Medical Devices.
31. SG1-N41R9:2005. Essential principles of safety and performance of Medical Devices.
32. SG5/N1R8:2007. Clinical evidence-key definitions and concepts.
33. SG5/N2R8:2007. Clinical evaluation,[D.M. n° 162 15 of July 1997: Fulfillment of European Community guidelines concerning Good Clinical Practice for clinical investigations execution involving drugs], (Italian).
